# Asymmetric Function of Theta and Gamma Activity in Syllable Processing: An Intra-Cortical Study

**DOI:** 10.3389/fpsyg.2012.00248

**Published:** 2012-07-19

**Authors:** Benjamin Morillon, Catherine Liégeois-Chauvel, Luc H. Arnal, Christian-G. Bénar, Anne-Lise Giraud

**Affiliations:** ^1^INSERM U960 – Ecole Normale SupérieureParis, France; ^2^INSERM U1106 – Institut de Neurosciences des systèmes, Aix-Marseille UniversitéMarseille, France

**Keywords:** intra-cortical, auditory, oscillation, asymmetric sampling, theta, gamma

## Abstract

Low-gamma (25–45 Hz) and theta (4–8 Hz) oscillations are proposed to underpin the integration of phonemic and syllabic information, respectively. How these two scales of analysis split functions across hemispheres is unclear. We analyzed cortical responses from an epileptic patient with a rare *bilateral* electrode implantation (stereotactic EEG) in primary (A1/BA41 and A2/BA42) and association auditory cortices (BA22). Using time-frequency analyses, we confirmed the dominance of a 5–6 Hz theta activity in right and of a low-gamma (25–45 Hz) activity in left primary auditory cortices (A1/A2), during both resting state and syllable processing. We further detected high-theta (7–8 Hz) resting activity in left primary, but also associative auditory regions. In left BA22, its phase correlated with high-gamma induced power. Such a hierarchical relationship across theta and gamma frequency bands (theta/gamma phase-amplitude coupling) could index the process by which the neural code shifts from stimulus feature- to phonological-encoding, and is associated with the transition from evoked to induced power responses. These data suggest that theta and gamma activity in right and left auditory cortices bear different functions. They support a scheme where slow parsing of the acoustic information dominates in right hemisphere at a syllabic (5–6 Hz) rate, and left auditory cortex exhibits a more complex cascade of oscillations, reflecting the possible extraction of transient acoustic cues at a fast (~25–45 Hz) rate, subsequently integrated at a slower, e.g., *syllabic* one. Slow oscillations could functionally participate to speech processing by structuring gamma activity in left BA22, where abstract percepts emerge.

## Introduction

Low-gamma (25–45 Hz) and theta (4–8 Hz) oscillations could under pin the chunking of continuous speech into phonemic and syllabic segments, respectively (Poeppel, 2003; Giraud and Poeppel, 2012). These two rhythms tend to be asymmetric at rest in Heschl’s gyrus, with theta dominating in right and gamma in left auditory cortex (Giraud et al., 2007; Morillon et al., 2010). Asymmetric endogenous oscillatory activity is compatible with distinct integration properties in right and left auditory cortices, because they could drive alternating low- and high-neuronal excitability states at different time-scales (Schroeder et al., 2008). This functional asymmetry is a plausible neurophysiological substrate of the greater sensitivity of the left auditory cortex to short sound segments and brief speech features (Jamison et al., 2006; Obleser et al., 2008), and of the greater sensitivity of the right one to slower acoustic fluctuations and larger steady speech signals such as vowels and syllables (Boemio et al., 2005; Abrams et al., 2008; Telkemeyer et al., 2009).

Given the spatial distribution of theta and gamma oscillations across right and left auditory cortices, it is unclear how these two rhythms interact in speech analysis. It would be counterintuitive to assume that information from both hemispheres needs to be integrated for leftward phonological analysis. While, in the right hemisphere, syllabic chunking at theta rate seems sufficient for processing paralinguistic information (prosody, speaker recognition…), speech could be analyzed at least at two time-scales by the *left auditory cortex*, with the embedding of phonemic- into syllabic-, up to word- or sentence-sized temporal frames (Giraud and Poeppel, 2012). From a computational perspective, phonemic chunks could be sampled at gamma rate, before being integrated at a slower time-constant, corresponding to syllabic-sized chunks, sampled at a theta rate. This concept entails the hypothesis that theta activity in left auditory cortex plays a specific integrative role. We thus sought to address the functional relevance of gamma and theta activity in auditory regions, probing in particular how they functionally interact.

Theta activity is present at rest in the left anterior temporal cortex, a location compatible with a possible integrative auditory function (Giraud et al., 2007). Our specific hypothesis is that theta activity in *right* auditory cortex plays a basic sampling function where it chunks speech signals into segments of about 100 ms [half a (~5 Hz) theta cycle]. This presumably does not permit to grasp all phonetic details, but allows for computations on formants to infer vowel categories and speaker identity, for instance. By contrast, theta rhythm in *left* auditory cortex could integrate information that has already been finely parsed at gamma rate. With nearly the same (~100 ms) integration window, the function of left theta could dramatically differ from that of the right theta, reflecting integrative properties based on a first-order detailed phonetic analysis (Giraud et al., 2007).

**Figure 1:**
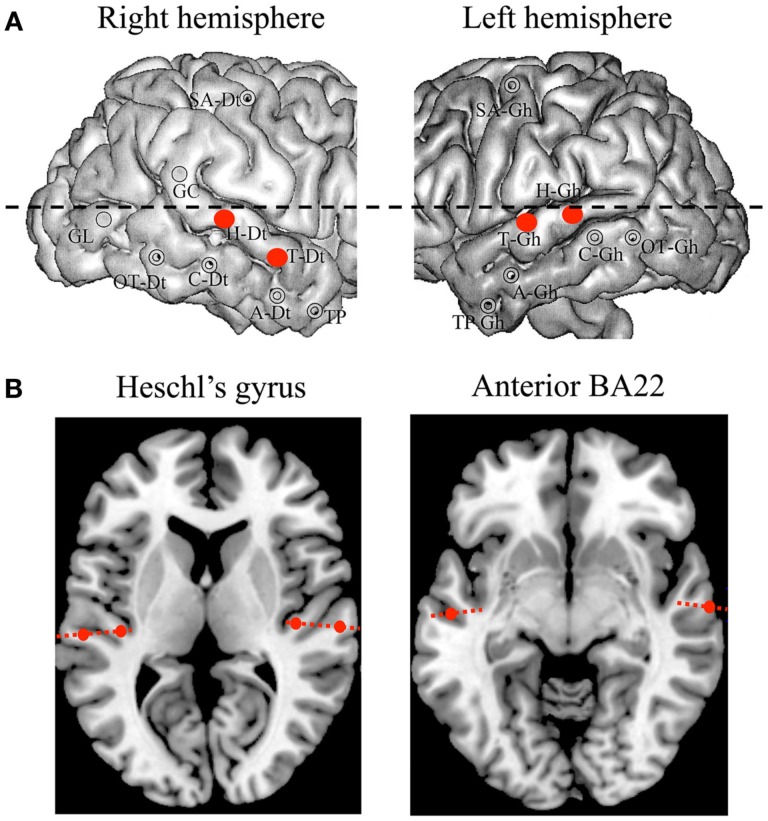
**Electrodes and contacts position**. **(A)** Implantation map, with the four auditory electrodes of interest highlighted in red (Heschl’s gyrus and anterior part of BA22, bilaterally). **(B)** Detail of anatomical position for each contact. The six contacts of interest, covering A1 and A2 (left panel), and BA22 (right panel), are magnified.

**Figure 2:**
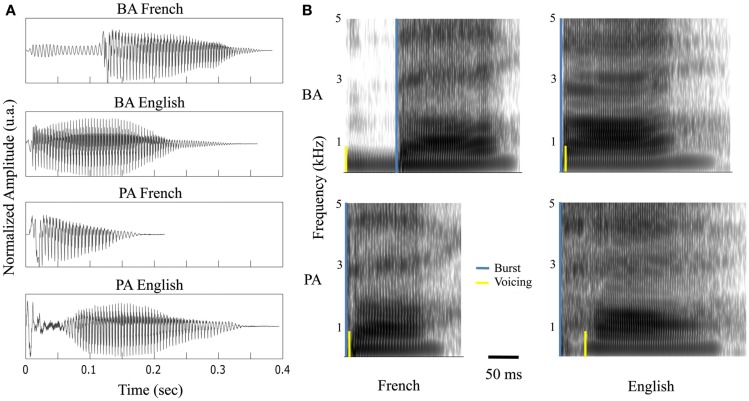
**Stimuli**. **(A)** Waveform and **(B)** spectrogram of the four syllables: /ba/ and /pa/, uttered by a French or English female.

Addressing such detailed functions of gamma and theta rhythms along the auditory cortex hierarchy simultaneously in *both* right and left auditory cortices is hardly conceivable in humans. Here, however, we had the unique opportunity to use stereotactic EEG (SEEG) recordings performed in an epileptic patient who carried, for clinical purposes, depth electrodes in both auditory cortices. These electrodes were positioned at three key locations (Figure 1): primary auditory cortex (A1), secondary auditory cortex (A2), and associative auditory cortex (anterior BA22); as assessed anatomically, and for A1, also functionally based on the shape and latency of early evoked responses to brief tones (Liegeois-Chauvel et al., 1991, 1994). Here, we analyzed data acquired both at rest and while the patient listened to different syllables (Figure 2), to investigate spontaneous and speech-related (evoked or induced) oscillatory patterns over three locations of the speech processing hierarchy.

## Materials and Methods

### Subject

A single patient participated in this study. Her native language was French. She suffered from drug-resistant partial epilepsy and was implanted for pre-surgical investigation with chronic depth electrodes (SEEG) in right and left auditory cortices; i.e., *Heschl’s gyrus* (primary and secondary auditory cortex, i.e., A1 and A2) and the anterior part of the *superior temporal gyrus* (STG, BA22), together with other cortical structures of no interest for the study (Figure 1). She provided informed consent to the protocol, which was approved of by the institutional review board of the French Institute of Health. Neuropsychological assessment indicated that she had intact language functions, with only superficial working memory and word fluency deficits. Brainstem evoked potentials and pure tone audiograms carried out before SEEG indicated intact cochlear and brainstem auditory functions. Analysis of SEEG concluded that the epileptogenic zone was located outside the regions analyzed here.

### Electrophysiological recordings

#### Sessions and stimuli

Data acquisition was divided in four sessions: (i) A *functional mapping session* during which 30 ms *pure tone* bursts (with a 0.3 ms rise and decay time) were presented binaurally 108 times, at 750 Hz, with an ISI of 1030 (±200) ms. (ii) A *resting state* session (eyes open) of 2.5 min. (iii) A *syllables processing session* comprising four *syllables*: /ba/ and /pa/ pronounced by an English or French female (Figure 2) and presented binaurally or monaurally in both ears, all in equal proportions, 501 times in total with an ISI of 1030 (±200) ms. For monaurally presented material, we analyzed contra-lateral responses. (iv) A *frequency-tagging session*, by use of a 1-s white noise modulated sinusoidally in amplitude at 8 Hz (i.e., *AM sound*), presented binaurally 58 times, with an ISI of 1064 ms.

#### Acquisition

Stimuli were presented through headphones in a pseudo-randomized order at a 22 kHz rate using E-prime 1.1 (Psychology Software Tools Inc., Pittsburgh, PA, USA). The patient was instructed to listen passively and concentrate on what she heard. SEEG recordings were monopolar, with each contact of a given depth electrode referenced to an extra-dural lead using acquisition software and a 128-channel SynAmps EEG amplification system from NeuroScan Labs (Neurosoft Inc.,). During the acquisition, the EEG signal was high-pass filtered at 0.5 Hz and amplified with an anti-aliasing filter at 200 Hz (temporal resolution of 1 ms and amplitude resolution of 1 μV).

### Anatomo-functional definition of contacts position

The stereotactic method was based on the co-registration of the patient’s MRI with the stereotactic angiogram, to prevent injury to brain vessels. Multi-lead electrodes (0.8 mm diameter, 10 or 15 contacts of 2 mm length each with 1.5 mm spacing between contacts) were orthogonally introduced in the stereotactic space (Szikla et al., 1977; Talairach, 1988). The anatomical position of each contact was then identified on the basis of (i) an axial scanner image acquired before the removal of electrodes, and (ii) an MRI scan performed after the removal of electrodes (Liegeois-Chauvel et al., 1991; Figure 1).

**Figure 3:**
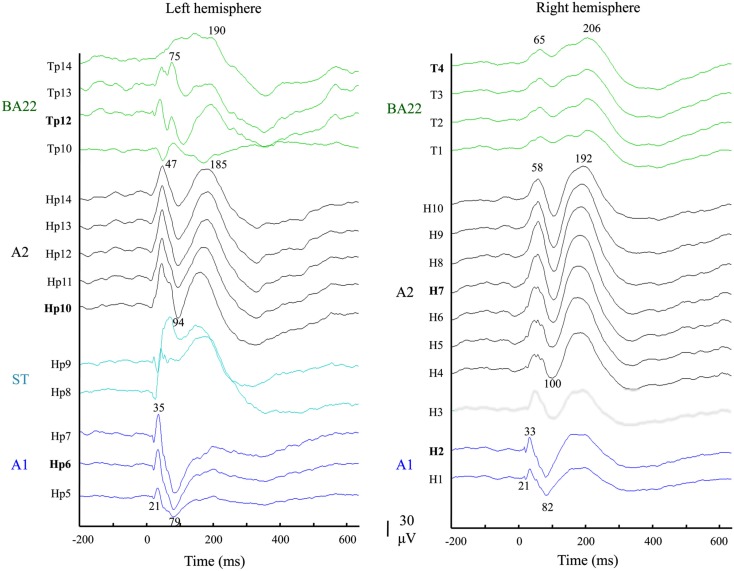
**Functional definition of the auditory territories**. Auditory evoked potentials in response to 750 Hz tone bursts recorded from the 2 × 14 responsive contacts: 10 continuous leads of electrodes implanted in Heschl’s gyri (Hp/left and H/right), and four in the anterior part of STG (BA22; Tp, and T; green traces). Electrodes Hp and H recorded activity in successive areas: A1 (blue traces), the sulcus transversus (ST; cyan traces in left hemisphere) and A2 (black traces). These were categorized according to their latency responses (see Materials and Methods).

Auditory evoked potentials (AEPs) measured in response to pure tones were used to functionally delineate the different auditory areas, and to select the right electrodes. AEP were averaged over trials, after epoching (−200 to 635 ms) and taking the −150 to −50 ms pre-stimulus time period as baseline. All contacts (25 out of 55) that elicited no significant responses (<40 μV) were discarded. In a second step A1 was functionally defined based on the presence of early P20/N30 components (Liegeois-Chauvel et al., 1991, 1994; Figure 3). These responses were located in the medial and intermediate part of Heschl’s gyrus. A2 is located in the lateral part of Heschl’s gyrus in the right hemisphere and in the Planum Temporale in the left hemisphere. Third, for each of the six functional areas (A1, A2, and BA22 in left and right hemispheres), the most responsive contact (Hp6, H2; Hp10, H7; Tp12, T4) was selected for the following analyses.

### Preprocessing

Data were analyzed in MATLAB (The MathWorks) with homemade scripts, EEGlab v.8 (sccn.ucsd.edu/eeglab) for data extraction, and in-house Fast_tf v4.5 software (cogimage.dsi.cnrs.fr/logiciels/) for time-frequency (TF) analysis.

#### Artifact rejection

Sessions with syllables, AM sounds, and pure tones were divided into epoch segments, including a 200 ms baseline prior to stimulus onset. Epochs including signals that *deviated* from the average response of all the trials were discarded. To do so, we computed the correlation between each single trial and the average response, and rejected the 15% trials with the lowest Pearson’s correlation value. We set this conservative rejection criterion and validated the approach on the basis of visual inspection of the signal.

#### Referencing

All contacts were subtracted from a common reference signal that corresponded to the average response of the least responsive (mesial or lateral) contact of each 4-electrode. This resulted in attenuating global noise (ex: 50 Hz ambient electric field) in a similar way for all contacts.

#### Time-frequency analysis

A TF wavelet transform was applied to the resting state session, together with each trial of the syllable and AM sound sessions, using a family of complex Morlet wavelets (*m* = 7; and *m* = 20 for the continuous resting state session), resulting in an estimate of oscillatory power and phase at each time point and each frequency, with a 0.5 Hz resolution below 20 and 1 Hz above. Importantly, the TF resolution of the wavelets was frequency dependent (for *m* = 7, at 7 Hz: σ = 150 ms, 1 Hz; at 35 Hz: σ = 30 ms, 5 Hz). Frequency spectrum of analysis was restricted to 1–45 Hz for *resting state*, i.e., the most energetic part (and below the French electric artifact); and 3–150 Hz for *syllable* and *AM sounds*, the lowest frequency being defined by the shortest trial duration.

### Power analysis

#### Syllable and AM sound sessions

After transformation of the TF power data in decimal logarithmic units (so that they follow a Gaussian distribution), the 100 ms period preceding each trial (–150 to −50 ms relative to stimulus onset) was used as baseline (50 ms were excluded to avoid border effects). We only present analyses based on power modulations (in dB), defined at each time and frequency data-point as the increase of signal power relative to baseline in decimal logarithmic units. For the AM sound data, we moreover extracted the 1-s-averaged power response at 8 Hz, matching the stimulus in both frequency and duration.

#### Resting state

Time-Frequency power data were averaged over time in 21 non-overlapping 10 s segments after transformation in decimal logarithmic unit (Gaussian normalization). Inside the 1–20 Hz range, deviations from the 1/*f*  logarithmic trend were estimated by fitting each of the 21 segments (for each of the six contacts of interest) to a function *a*·*x^b^*. This was accomplished by converting the spectrum to log–log coordinates and fitting a line [the slope of which corresponds to *b* = −1.4 (±0.04)] using a least squares estimate.

### Phase-locking value

The evoked activity was computed by mean of the phase-locking value (PLV) for each TF data-point, according to the relation:

$$\text{PLV}=\frac{1}{n}\left|\overset{n}{\underset{t=1}{\sum }}\text{ex}{\text{p}}^{i\phi t,f}\right|$$

Where *n* is the number of trials and Φ the instantaneous phase. PLV measures thus the variability across trials and equates 1 if the trials are perfectly phase-locked (i.e., time-locked to the stimulus) and 0 if they are totally unsynchronized. Components present in the global power response and absent in the PLV response were considered as induced.

### Phase-amplitude coupling analysis

Cross-frequency dependencies were studied under the phase-amplitude coupling framework by use of the *modulation index (MI;* Tort et al., 2008). It is based on a normalized entropy measure of the high-frequency power (*f*_a_) according to low-frequency phase (*f*_p_) signal. For each frequency power *f*_a_ (3–150 Hz) and contact, the phases Φ*_t,n_*(*f*_a_) are binned into eighteen 20° intervals across trial and time dimensions, and the mean of (*f*_a_) over each phase bin is calculated. Note that we normalized raw- (non-logarithmic) power (*f*_a_) between 0 and 1 for each trial and frequency.

### Statistical validation

Log-transformed power data were normally distributed, allowing for the use of standard parametric tests to assess the statistical significance of observed effects.

#### Asymmetric power responses

Inter-hemispheric power asymmetries where quantified with *t*-test statistics at *p* < 0.01. Moreover, we applied a FDR correction (over the frequency dimension) when analyzing the whole frequency spectrum (resting state data). For asymmetry quantification in the TF domain (syllables responses) we applied a FDR correction (over both dimensions at once), and discard clusters <500 data-points.

#### Asymmetric PLV

For each TF data-point we compared the correlation coefficients (PLV) obtained in right and left hemispheres. PLV were Fisher transformed and used to compute the *Z* statistics, according to the relation:

$$Z=\frac{\left|\text{PL}{\text{V}}_{\text{left}}-\left.\text{PL}{\text{V}}_{\text{right}}\right|\right.}{\sqrt{\frac{1}{n_{\text{left}}-3}+\frac{1}{n_{\text{right}}-3}}}$$

Where *n* corresponds to the number of trials (*n*_left_ = *n*_right_ = 486). We considered as significant *Z*-values > 2.58 (corresponding to *p* < 0.01 uncorrected; i.e., an absolute difference of PLV > 0.166), and further discard clusters <500 data-points.

#### Cross-syllable effects

Computation of asymmetric power responses (and PLV) allowed us to highlight different components (TF clusters) of interest, either left- or right-dominant. For each component, cross-syllable differences were assessed through repeated-measures ANOVAs main effect, at *p* < 0.05, while controlling for possible side-of-presentation (monaural vs. binaural) effects.

#### Phase-amplitude coupling statistics

They were performed via non-parametric cluster analysis (Maris et al., 2007), through permutation (bootstrap), by computing 200 times the MI with randomization of the trial dimension. For each phase- and power-frequency, the value of the 99th percentile (corresponding to *p* < 0.01) was subtracted from the original MI in order to plot only significant results.

## Results

### Resting state and 8 Hz AM sound stimulation

**Figure 4:**
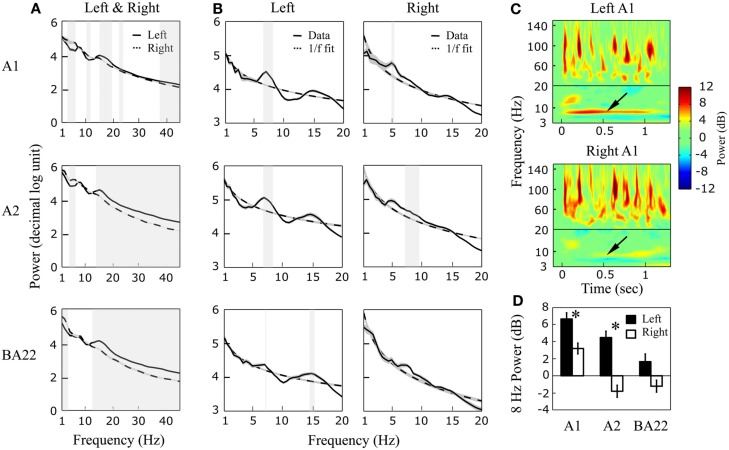
**Resting state and 8 Hz AM sound stimulation**. **(A)** 1–45 Hz power spectrum of a 2.5-min resting state period, for left and right auditory areas (A1, A2, and BA22). Left power dominance is usually visible above ~13 Hz (significant inter-hemispheric differences are highlighted in gray; paired *t*-test, *p* < 0.01 FDR corrected). **(B)** Detail of the 1–20 Hz range with 1/*f* fitting curves (dashed lines). Note a global peak at 7.5 (and 15) Hz in left auditory cortex, and at 5.5 Hz in the right one. Significant deviations from the 1/*f* fit are highlighted in gray (unpaired *t*-test, *p* ≤ 0.01 FDR corrected). **(C)** Time-frequency power response to a 1-s 8 Hz AM sound, in left and right A1. **(D)** Extraction of the 1-s-averaged power response at 8 Hz (see arrows in C) in the six regions of interest. *Indicates significant inter-hemispheric differences (paired *t*-test, *p* < 0.01).

Power spectra of resting state data were computed between 1 and 45 Hz (Figure 4A). Significant deviations from the 1/*f* pattern were visible, indicating the presence of intrinsic oscillations in low frequency bands (Figure 4B). In the left hemisphere, oscillatory activity was present in all three-regions (A1, A2, and BA22) and peaked at 7.5 Hz, with an additional peak at 15 Hz (significant in BA22; possibly reflecting theta harmonic). In right auditory cortex a peak was visible in A1 and A2 at 5.5 Hz. Theta asymmetry was thus observed in both spectral and spatial domains, with a faster theta (7–8 vs. 5–6 Hz) reaching higher hierarchical levels (i.e., BA22) in the left hemisphere. Stimulation with a 1-s 8 Hz AM sound confirmed asymmetric theta preferences, with a larger oscillatory entrainment at 8 Hz in left A1 and A2 (Figures 4C,D) relative to their right counterpart. In the gamma range, we could not detect significant power deviations from the 1/*f* trend, possibly because of averaging (gamma activity was visible in single trials, though very variable). The overall power was significantly higher in left than right hemisphere from ~13 Hz and above (mainly in A2 and BA22), including beta and gamma ranges, and higher in right than left within the low-frequency range (<12 Hz; Figure 4A).

### Syllables processing: Global power and PLV

**Figure 5:**
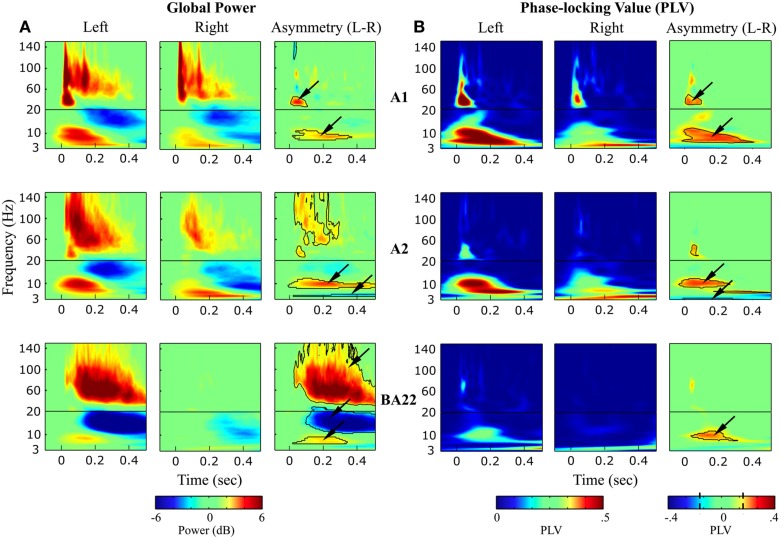
**Syllables processing**. **(A)** Time-Frequency (global) power response and **(B)** phase-locking value (PLV; reflecting evoked activity) averaged over the four syllables, in left and right auditory areas (A1, A2, and BA22). Right (third and sixth) panels: Asymmetric (left-right) oscillatory components, with contour paired *t*-test statistics threshold at (A): *p* < 0.01, FDR corrected, cluster extent > 500 data-points; and (B): Z > 2.58, i.e., *p* < 0.01 uncorrected, cluster extent >500 data-points. Note that (1) Asymmetric activity is organized in frequency-specific clusters of activity. (2) High-gamma and beta responses (only visible in the global power response) correspond to induced activity.

Time-Frequency plots of power modulation in response to syllables perception indicated transient and sustained oscillatory components within different frequency bands (Figure 5A). In A1, high-gamma transient onset responses were symmetrical in left and right hemisphere, indicating that both electrodes were equally capturing neuronal activity. The left-lateralization of sustained high-gamma activity (>50 Hz) increased along the cortical hierarchy, co-occurring with a deactivation in the beta band (~12–20 Hz). Lower-frequency responses had distinct response profiles in right and left hemispheres, in line with their hypothesized roles in speech parsing. Quantification of the significantly asymmetric responses (Figure 5A, right-panel) revealed that low-gamma (25–45 Hz) transient responses were left-dominant in Heschl’s gyrus only (maximal in A1). High-theta (~7.5 Hz) responses were left-dominant within all three sampled cortical areas. Low-theta (~5.5 Hz) responses were right-dominant and maximal in A2.

Comparing global power and PLV responses (Figures 5A,B) allowed us to infer the nature of the oscillatory responses. They could be categorized as *evoked* (if represented in both the power and PLV responses; i.e., time-locked to the stimulus) or *induced* (only present in the global power response) types. Activity split rather unambiguously between evoked or induced activity. Unsurprisingly, responses tended to be less stimulus-driven in more associative areas. While low-gamma responses in A1 corresponded to a transient response phase-locked to the stimulus, high-gamma response in left BA22 was purely induced. All induced activity was in the high-gamma and beta range, reflecting internal processes, independent of stimulus tracking. By contrast, evoked activity was below 50 Hz, including low-gamma, high-theta, and low-theta responses, with identical significant asymmetric response profiles observed in global power and PLV (right-panels in Figures 5A,B).

### Cross-syllable effects

**Figure 6:**
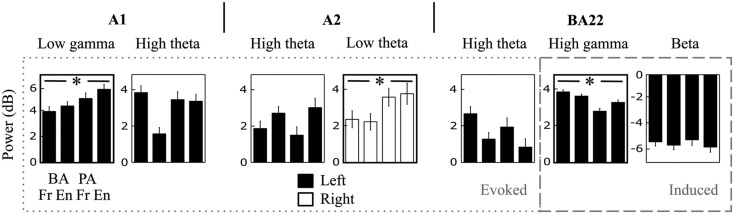
**Cross-syllable effects**. Detail of the power response to each syllable, for each (left- or right-hemispheric) time-frequency component of interest (i.e., the significant asymmetric effects; see arrows in Figure 5A). * Indicates main effect ANOVAs at *p* < 0.05.

The effects described above were observed in each of the four studied syllables, we thus presented patterns averaged across all syllables (Figure 5). However, some of the *components* of interest (i.e., those showing a significant hemispheric dominance) did present significant cross-syllable differences, reflecting functional specificities. Of the seven components (arrows in Figure 5A), only the low-gamma evoked response in left A1, the low-theta evoked response in right A2, and the high-gamma induced response in left BA22 showed a syllabic effect (Figure 6). The variability of the high-theta components was explained by the side-of-presentation, which was modeled as a variable of no interest.

### Theta/gamma cross-frequency phase-amplitude coupling

**Figure 7:**
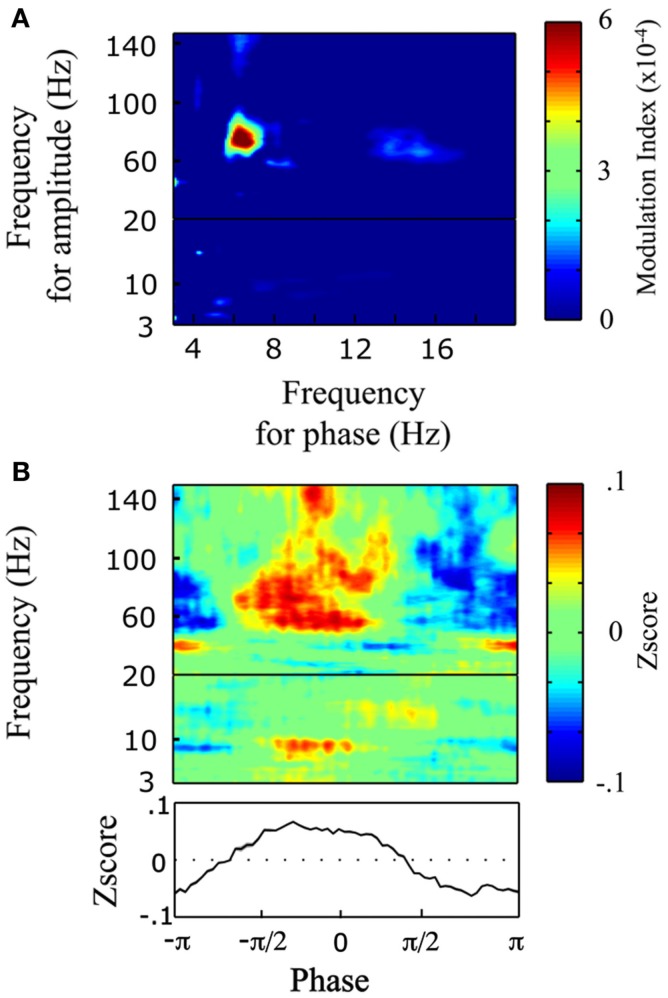
**Phase-amplitude coupling in left BA22**. **(A)** Comodulogram between low-frequency phase (3–20 Hz) and power (3–150 Hz). Positive modulation index values indicate significant coupling (*p* < 0.01, bootstrap). **(B)** Power (3–150 Hz) distribution over the phases of theta (7.5 Hz). *Bottom panel:* averaged *Z*-score over high-gamma (60–100 Hz) power for theta phase angle.

To understand the mechanism underlying transition from evoked (stimulus phase-locked) to induced power responses, and the associated process of abstraction of phonological representations, we tested for cross-frequency relation by measuring phase-amplitude coupling, i.e., the interaction between low-frequency phase and high-frequency power (Tort et al., 2008). We focused our analysis on left BA22, the region with maximal induced response. Our statistical approach (shuffled trials) allowed us to highlight trial-specific co-modulations, i.e., to exploit inter-trial variability. We observed a significant co-modulation between high-theta phase and high-gamma power (Figure 7A). This coupling coincided approximately with the peak frequency of the evoked activity (theta at 7.5 Hz; lower-panel on Figure 5B) and with the peak frequency of the induced activity (high-gamma at 64 Hz; lower-panel on Figure 5A). Finally, the power response was maximal on the rising phase of the theta cycle, just before the peak (Figure 7B).

## Discussion

### Lateralized theta components: Frequency-by-hemisphere interaction

The current results obtained in a patient who had depth electrodes implanted in bilateral auditory cortices offer precious insight into the variety of expression and function of oscillatory activity, even though they can only be generalized with caution. For each hemisphere three regions were investigated. AsA1 an A2 showed similar responses, the three studied regions likely represent two rather than three hierarchical stages, which is consistent with previous findings (Liegeois-Chauvel et al., 1999). We could confirm that resting theta activity dominates at 5–6 Hz in right A1/A2 (Figure 4B). Although we did not detect reliable gamma peaks at rest, global power in the gamma range was higher in left than right auditory cortex (Figure 4A). These results are globally consistent with the *Asymmetric Sampling in Time* theory (Poeppel, 2003) that stipulates gamma dominance in left auditory cortex and theta dominance in right auditory cortex, but also offer interesting complementary findings.

Additional on-going activity was detected in left auditory cortices in the theta range, though at a different, slightly higher, frequency (7.5 Hz) than in right auditory cortex. Unlike the right low-theta resting activity, this left high-theta activity persisted in BA22, i.e., when moving up the hierarchy. Such a spectro-spatial double dissociation is so far in line with our assumption that theta rhythms could bear different functions in right and left hemisphere.

In agreement with theories suggesting that spontaneous activity is a hallmark of internal models and states (Sadaghiani et al., 2010), reflecting both experience and developmental programs (Berkes et al., 2011), resting state pattern of activity in the theta range was predictive of subsequent power modulations during sound processing. When auditory cortices were stimulated by an 8 Hz modulated sound, the entrainment was stronger in left than right A1/A2 (Figure 4C), presumably owing to the presence of the intrinsic 7.5 Hz activity. As high-gamma bursting activity was more pronounced in right than left auditory cortex (see also Liegeois-Chauvel, 2004), left theta activity during auditory stimulation likely results from a true interaction between on-going sustained low-frequency neural activity and the stimulus, rather than merely reflecting the low-pass components of gamma bursting responses. This is an important piece of evidence for a distinct role of left and right theta rhythms in speech processing.

### Specific roles of evoked theta/gamma activity

To more specifically address the function of theta and gamma activity in right and left auditory cortices, we examined TF activity in response to syllables. We confirmed that resting state patterns were predictive of responses to sensory stimulation, by finding asymmetric components in the low-gamma (left), high-theta (left), and low-theta (right) ranges (right-panels in Figures 5A,B). Second, by looking at global power that reflects both evoked and induced components, and PLV indexing evoked (i.e., time-locked) activity, we observed that all the lower-frequency components (<50 Hz; except beta activity) were time-locked to the stimulus (i.e., evoked; see also Steinschneider et al., 2007), in accordance with a speech-tracking role (Luo and Poeppel, 2007; Luo et al., 2010). Note however that even these “so-called” evoked responses presented some phase variability over trials (PLV < 1).

By analyzing cross-syllable power responses across seven TF components showing a hemispheric dominance, we found that only three showed cross-syllable power differences: (left A1, evoked) low-gamma, (right A2, evoked) low-theta, and (left BA22, induced) high-gamma (Figure 6). Such a syllable effect has different implications depending on whether it is observed on evoked or induced components [see also Induced Responses (in Left BA22)], as time-locked evoked activity likely underpins acoustic features tracking, whereas induced activity likely reflect endogenous processing. Our findings hence suggest that left low-gamma and right low-theta oscillations underlie speech-tracking. Left auditory cortex could extract transient acoustic, phonemic features at fast rate (e.g., Voice Onset Time; Steinschneider, 2004). Typically, gamma adequately encodes acoustic patterns lasting around 50 ms by two to three cycles (Hoonhorst et al., 2009; Shamir et al., 2009). Its right counterpart would co-vary with slower acoustic features, e.g., the syllabic rate.

### Induced responses (in left BA22)

While evoked responses showed equal asymmetry in the three investigated regions (Figure 5B right panel), induced responses appeared symmetric in A1, and became dramatically left-dominant from A2 onward. Induced activity was composed of two main components: high-gamma and beta (Figure 5A). Given that the set of stimuli was repeated, hence in part predictable, joint high-gamma power increase and beta power decrease is compatible with the role of beta activity in top-down message passing (Arnal et al., 2011). However, only high-gamma showed cross-syllable variability (Figure 6). This could reflect that beta driven predictions were not syllable specific, but that any syllable could occur. Conversely, the variability in gamma power might be related to abstract percept emergence, as claimed by Griffiths et al. (2010).

That in left BA22 the phase of evoked high-theta was related to induced-gamma power suggests that this high-theta activity could temporally structure high-gamma activity, and accompany the transition from acoustic-feature tracking to abstract (phonological) processing. That this co-modulation was trial-specific speaks against a direct relationship between acoustic dynamics and high-gamma power (Zion-Golumbic and Schroeder, 2012). Gamma activity was stronger during the rising part of the theta cycle, and it could be that one theta cycle signals the temporal limits of the event that is to be processed. Our observation suggests that a radical code transformation occurs between A2 and BA22 in syllable representation, with the apparition of induced (gamma) activity that is suggestive of a sustained spiking mode, compatible with new rate coding principles (Singer, 1999). Unfortunately, the current data do not provide additional clues about the nature of the neural code transformation.

### High-theta function

Our data show two different theta rhythms in bilateral auditory cortices, which is a novel finding requiring further confirmation in healthy participants or more epileptic patients. Left-hemispheric theta response during speech processing was already observed in other studies, but its specific role and frequency-specific asymmetric distribution during speech processing has never been addressed (Canolty et al., 2007; Luo and Poeppel, 2007). The spatial location of left high-theta activity supports the idea that it could underlie more advanced processing in left than right hemisphere, i.e., integration rather than parsing (Giraud et al., 2007). However our study did not allow for disentangling between the two following hypotheses. While high-theta and low-gamma responses in A1 could reflect *simultaneous* parsing at syllabic and phonemic rates, respectively, these two components could also reflect *successive* processing stages, with the information parsed at low-gamma rate being integrated in a *syllabic-like* representation at high-theta rate. At any rate, high-theta activity is essentially *evoked* by the syllables acoustic structure. That high-theta activity is present across hierarchical stages (1) suggests that it could integrate and coordinate different processing stages (integrate phonemic information in A1 and drive the abstract processing in BA22, via phase-amplitude coupling), and (2) is compatible with embedded processing stages of phonemic, syllabic, up to the word- or sentence-level, the latter requiring computations on large spatial and temporal scales. This interpretation entails the testable hypothesis of an evoked delta response during sentence perception present in a broader spatial scale, i.e., all over the associative auditory cortex.

That theta rate is different in left- and right-auditory cortices could reflect that right low-theta underpins first order syllabic parsing (Greenberg, 1998), and left high-theta related to a second order integration after low-gamma sampling. This idea is compatible with data showing that speech envelope (syllabic dynamics) is better represented in right than left auditory cortices (Abrams et al., 2008).

### Conclusion

The current results are globally consistent with previous observations made with other methodologies in humans (MEG, EEG/fMRI; Giraud et al., 2007; Morillon et al., 2010). All methods converge on the existence of an intrinsic asymmetry across auditory cortices based on a distinct dominant oscillatory activity type (gamma/left, theta/right). This intrinsic asymmetry could contribute to the widely established functional left dominance in language processing. The current data show that responses to stimulus reflect the intrinsic properties of auditory cortices. The early and rather subtle functional asymmetry in A1 and A2 presumably determines the profound functional splitting across left and right auditory cortices, because down-stream processing is conditioned by the properties of the first integrative stages (e.g., down-sampling rate, relevance of the extracted features for linguistic vs. vocal or paralinguistic processing). The current results also suggest that theta activity that is present (although non-dominant) in left auditory cortex participate in second-order speech processing computations, and presumably in the shift from a temporal/analog to an abstract code.

## Conflict of Interest Statement

The authors declare that the research was conducted in the absence of any commercial or financial relationships that could be construed as a potential conflict of interest.
